# Social workers’ formal and informal leadership in interprofessional primary care teams in Ontario, Canada

**DOI:** 10.1177/08404704231184582

**Published:** 2023-07-01

**Authors:** Rachelle Ashcroft, Nele Feryn, Simon Lam, Amina Hussain, Catherine Donnelly, Kavita Mehta, Jennifer Rayner, Deepy Sur, Keith Adamson, Peter Sheffield, Judith B. Brown

**Affiliations:** 1 152790 University of Toronto, Toronto, Ontario, Canada.; 2 26656 Ghent University, Ghent, Belgium.; 3 12363 Queen’s University, Kingston, Ontario, Canada.; 4 644711 Association of Family Health Teams of Ontario, Toronto, Ontario, Canada.; 5 Alliance for Healthier Communities, Toronto, Ontario, Canada.; 6 241838 Ontario Association of Social Workers, Toronto, Ontario, Canada.; 7 70384 Western University, London, Ontario, Canada.

## Abstract

The development of interprofessional teams in primary care presents opportunities for social workers to take on new leadership positions. This study seeks to describe how social workers engaged in leadership roles in primary care during the COVID-19 pandemic. A cross-sectional on-line survey was disseminated to primary care social workers across Ontario, Canada, with a total of 159 respondents. Most respondents engaged in informal leadership roles and showcased a range of leadership skills promoting team collaboration and consultations, along with adapting to virtual care transitions. Findings suggest there needs to be intentional cultivation of social work leaders through supportive environments and training. Social workers in primary care have leadership capacity and are providing leadership to their primary care teams through formal and informal means. The leadership potential of social workers in primary care teams, however, is being underutilized and can be further developed.

## Introduction

Leadership is an essential component of interprofessional healthcare settings.^1^ The development of primary care teams has brought leadership to the forefront, requiring coordination of professionals with diverse skills and backgrounds.^2,3^ Social workers have been integrated into interprofessional primary care teams to enhance access to a range of psychosocial and mental health services^4,5^ and to provide comprehensive care for individuals and communities with increasingly complex needs.^6^ Although social workers hold skills and capabilities to function in leadership roles, the extent that social workers are providing leadership within primary care team contexts is unknown.^7,8^

Leadership has been defined as having the ability to nurture an environment where all employees can contribute their full potential in support of the organization’s mission.^9^ In healthcare contexts, leadership typically includes the ability to identify priorities, provide strategic direction, and foster a commitment across multiple sectors and stakeholders to address the identified priorities.^10,11^ Leadership shapes organizational culture and climate, influences structures and processes, and informs practical aspects of organizational functioning.^12^ Relational leadership includes communicating regularly and clearly across various levels of an organization, acknowledging diverse opinions, sharing knowledge, and engaging meaningfully with others.^12^ The LEADS framework articulates five dimensions that represent the leadership competencies required for healthcare contexts^13^: (i) lead self, (ii) engage others, (iii) develop coalitions, (iv) system transformation, and (v) achieve results. Striving for a culture of distributed leadership, the LEADS framework also recognizes that all providers can exercise leadership when required.^13^ Similarly, leaderful practice is an approach that values collaboration and encourages all team members to collectively and concurrently participate in leadership.^14^

Leadership competencies are a critical aspect of effective social work practice in healthcare^12,15^ yet little is known about leadership pertaining to allied health professionals, including social workers, compared to other clinical providers in healthcare contexts.^7^ Despite the integration of social workers in primary care, there is limited research to understand social workers’ formal and informal leadership within these teams^16,17^ and an absence of literature on social work leadership in healthcare during times of crisis, such as the COVID-19 pandemic.^15^ To bridge this knowledge gap, our study sought to (i) describe social workers’ formal and informal leadership roles in primary care teams, and (ii) explain how social workers in primary care undertook leadership in response to needs arising during COVID-19.

## Methods

This study used a cross-sectional on-line survey design and disseminated using Qualtrics software. Two of the main interprofessional primary care team models in Ontario are Family Health Teams (FHTs)—which serve approximately three million Ontarians (or about one-quarter of the population) – and Community Health Centres (CHCs), a team-based model aimed at patients with complex health and social care needs.^18^ Many FHTs, CHCs, and other primary care teams in Ontario—such as Nurse Practitioner-Led Clinics (NPLCs)—include social workers.^5^

We obtained a convenience sample of social workers employed in primary care teams in Ontario, Canada, who completed a web-based survey in English. At the time of the survey, there were approximately 700 full-time equivalent social work roles in primary care in Ontario. We conducted the survey from April 4 to November 9, 2022. Recruitment consisted of e-mails to social workers at FHTs, NPLCs, CHCs, social work e-mail lists, and social media. Three community partners—the Association of Family Health Teams of Ontario, the Alliance for Healthier Communities, and the Ontario Association of Social Workers—shared study information with their members with aims to reach the majority of social workers employed in primary care at time of the survey. Participation in the survey was voluntary and without recruitment incentive.

The entire survey consisted of 45 items, included closed-ended and open-ended questions, and took approximately 15 minutes. Survey domains included (i) practice characteristics, (ii) background on role, (iii) primary care context, (iv) structure of practice, (v) improving the structure of practice, (vi) clinical activities, (vii) leadership, and viii) demographics. The focus of this article is on questions that specifically asked about leadership. Descriptive statistics on the closed-ended questions were collated using Qualtrics software.

## Results

A total of N = 159 social workers in primary care were included in this study. There was geographical representation across the five Ontario Health Regions: West (37%), East (20%), Central (16%), Toronto (14%), North (12%), and unknown (1%). Respondents worked in FHTs (52%), CHCs (35%), NPLCs (6%), and other primary care settings (7%), and held the formal job title of social worker (70%), mental health counsellor (22%), registered psychotherapist (8%), and other titles (10%). Most respondents worked in primary care settings where there were other social workers (87%). Table 1 provides an outline of the demographics of the respondents.

**Table 1.** Respondent demographics.

Characteristic	Number of participants (%)
Years in practice as a social worker	
Less than 1 year	3 (2%)
1 to 5 years	28 (18%)
6 to 10 years	25 (16%)
11 to 15 years	34 (21%)
16 to 20 years	20 (13%)
Over 20 years	41 (26%)
No response	8 (5%)
Years of working in current position	
Less than 1 year	27 (17%)
1 to 2 years	27 (17%)
3 to 4 years	37 (23%)
5 to 6 years	14 (9%)
7 to 8 years	9 (6%)
Over 8 years	37 (23%)
No response	8 (5%)
Educational background	
Master of social work	115 (72%)
Bachelor of social work	40 (25%)
Social service worker diploma	11 (7%)
Other	18 (11%)
No response	8 (5%)
Age (years)	
18 to 25	2 (1%)
26 to 40	44 (28%)
41 to 55	56 (35%)
56 to 70	25 (16%)
Prefer not to answer	3 (2%)
No response	29 (18%)
Racial or cultural background	
Caucasian	102 (64%)
First Nations	5 (3%)
South Asian	5 (3%)
Black	5 (3%)
Chinese	4 (3%)
Métis	3 (2%)
Latin American	2 (1%)
Inuk/Inuit	1 (1%)
Filipino	1 (1%)
Other	6 (4%)
Prefer not to answer	5 (3%)
No response	29 (18%)
Gender	
Female	108 (68%)
Male	18 (11%)
Non-binary	1 (1%)
Two-spirit	1 (1%)
Other	2 (1%)
No response	29 (18%)

### Leadership in practice

Most respondents reported facilitating team dynamics (84%) and providing direct patient consultation to other team members (75%). A third of respondents reported formally organizing or leading team meetings while 43% of respondents reported acting as a formal mentor or supervisor for other team members. Two-thirds of respondents participated in program development and evaluation activities. More than half of the respondents (56%) indicated using their leadership skills to influence decision-making processes. A few respondents (13%) indicated acting as representatives for local stakeholder groups outside of their direct primary care team.

#### Informal leadership

Many respondents (75%) stated that they informally provided leadership to their team members. They were mainly engaged in promoting team collaboration and team dynamics by way of guidance and supportive directions, providing consultation to other disciplines and engaging in program development and/or program evaluation activities. Some (34%) of the respondents who identified as informal leaders participated in organizing and leading team meetings. More than half (58%) of respondents with informal leadership roles reported directly influencing decision-making processes within their team. A similar number (57%) of respondents with informal leadership roles were involved in quality improvement initiatives. Some respondents (17%) indicated that they do not exhibit leadership, yet when asked about specific leadership activities, the majority still indicated conducting leadership activities. For example, three respondents (2%) indicated that they did not exhibit leadership yet reported acting as a formal representative for local stakeholder groups. A few (12%) of the informal leaders acted as representatives for local stakeholder groups.

#### Formal leadership

**Figure 1.**
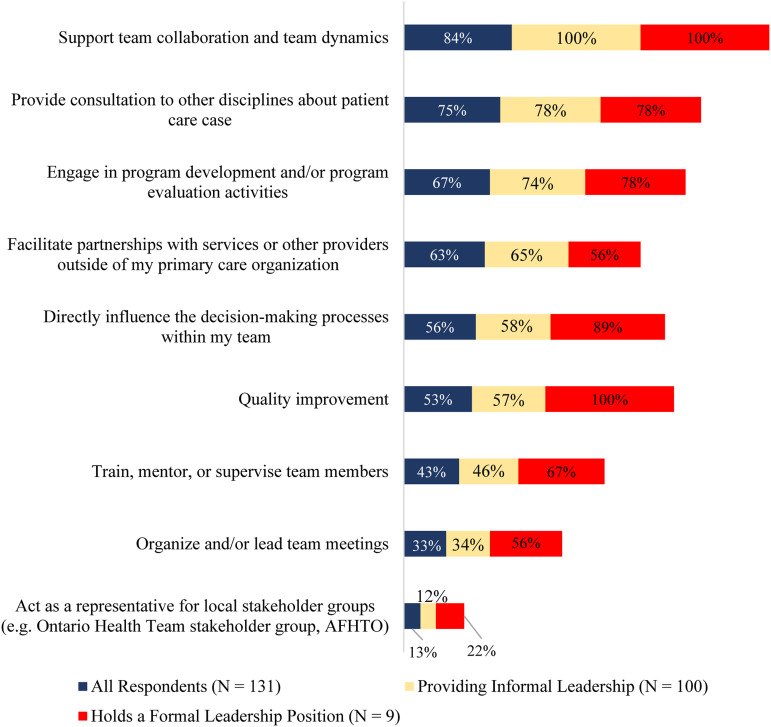
Types of leadership activities that social workers in primary care engaged in (n = 131).

While most respondents reported engaging in various leadership activities as part of their work, only a small percentage (7%) held a designated formal leadership role. Of the few respondents with formal leadership roles, most (89%) reported directly influencing decision-making processes within their team. All respondents with formal leadership roles engaged in quality improvement initiatives. Organizing and leading team meetings were more common among respondents identifying as formal leaders with over half (56%) doing so. A few (22%) of the formal leaders acted as a representative for local stakeholder groups. Figure 1 illustrates the types of leadership activities that social workers in primary care reported undertaking.

### Leadership during COVID-19

**Figure 2.**
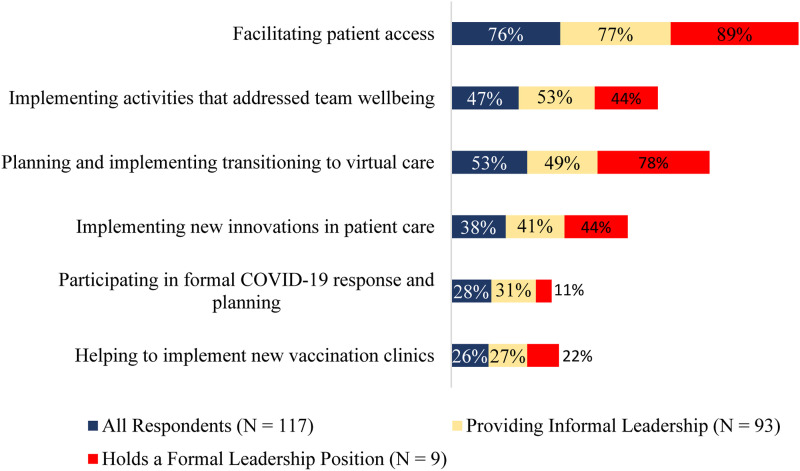
Types of leadership activities social workers in primary care engaged in related to the COVID-19 pandemic (n = 117).

The survey also explored the ways in which social workers provided leadership during the COVID-19 pandemic. Most respondents (76%) reported facilitating patients’ access to healthcare services with the majority (53%) noting involvement in planning and implementing virtual care for patients. Most (78%) of the formal leaders and less than half (49%) of the informal leaders were involved with the transition to virtual care. Some (38%) respondents reported having implemented new innovations for patient care, and nearly half (47%) of respondents conducted activities to enhance their team’s well-being. A minority (28%) of respondents indicated that they were involved in formal COVID-19 responses and planning, and over a quarter (26%) of respondents helped with delivering vaccination clinics. Figure 2 provides an overview of the types of leadership activities social workers undertook during the COVID-19 pandemic.

### Confidence and skills

Of the few respondents holding formal leadership roles, many (78%) received some form of supervision or guidance at work, and many (78%) of the formal leaders continued to seek out additional opportunities for supervision. In addition, some (44%) respondents with formal leadership roles cited education and training as key factors in maintaining their leadership skills. All respondents with a formal leadership position reported being fairly (44%) or very (56%) confident in providing leadership.

**Figure 3.**
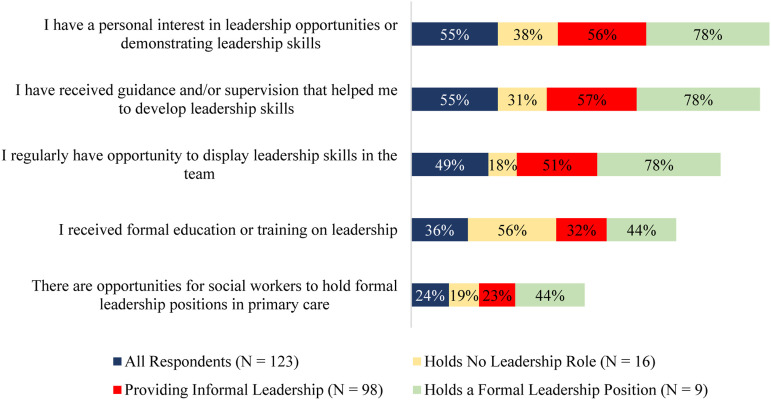
Existing supports for developing social workers’ capacity for leadership in primary care (n = 123).

Many (78%) respondents overall identified opportunities to demonstrate leadership skills on their team as important for developing effective leadership. All respondents, with the exception of one, identified education and supervision important for developing their future leadership capacity. Nearly all respondents, except for one, also reported that having more leadership opportunities in the workplace would be beneficial in developing their leadership capacity. In this regard, one respondent noted in an open-ended response: “*We are not seen outside of our mental health roles, when social workers offer much more than that.”* Figure 3 illustrates the existing leadership supports for social workers in primary care.

### Building capacity

Some (36%) respondents reported having received formal education or training in leadership, while many (65%) respondents reported that additional education and training would strengthen their leadership capacity. Only 10% of all respondents were not interested in enhancing their leadership skills. Respondents reported that receiving supervision and/or guidance (54%), accessing opportunities for formal leadership skills within a team (61%), and having opportunities for displaying leadership skills (59%) were factors that could strengthen their leadership capacity. In an open-text box that enabled respondents to identify any other additional supports for building capacity, respondents stated that a "*healthy environment,*" "*supportive manager,*" and "*management needs to allow this [being a leader] to happen.*” Another respondent wrote in the open-text box: “*There is no compensation for taking on leadership roles. It is an additional task to be completed along with your regular workload.”*

## Discussion

Leaders are needed to address healthcare system changes, respond to complex patient needs, and nurture collaboration in interprofessional primary care teams.^19^ Primary care teams are complex health systems requiring leadership approaches distinct from other healthcare sectors.^20^ The ability to lead a comprehensive care context, participate in collaborative decision-making, set expectations, clarify roles and responsibilities, facilitate conflict resolution, hold strong ethical frameworks, see a situation from various angles, and build consensus are demonstrative of fundamental leadership needs in interprofessional primary care teams.^21^ While reports on leadership in primary care largely focused on family physicians and administrative leadership roles,^19,22,23,24^ visibility of leadership from non-medical health professionals in primary care—such as social workers—is limited^7^ despite the need for leadership representing interprofessional perspectives to facilitate team functioning.^21^

We conducted a cross-sectional on-line survey to describe the leadership roles and activities of social workers in primary care in Ontario, Canada. Social workers have leadership capacity that is needed during this time of crisis due to worker shortages in primary care.^25,26^ While social workers in primary care held formal and informal leadership roles, understanding these leadership roles has been increasingly important to ensure alignment with the needs of the changing contexts of service delivery systems.^27,28,29^ Social workers in healthcare settings have historically provided leadership on social care, the social determinants of health, and structural inequalities like racism.^2,30^ This expertise aligns and enhances primary care’s foundational aims and commitments to equity, patient-centredness, and comprehensive care.^31^

Our study highlights that most social workers in primary care are engaging in leadership activities and demonstrating competencies that align with the LEADS framework competency dimensions: (i) lead self, (ii) engage others, (iii) develop coalitions, (iv) system transformation, and (v) achieve results.^13^ Social workers in our study engaged in leadership activities within their team that contributed to direct patient care and team functioning. Formally and informally, social workers provided leadership related to teamwork and collaboration which is essential for the functioning of interprofessional primary care teams^19^ and aligns with the core competency of engage others.^13^ Social workers mediate crisis, resolve conflict, and have an ability to lead and set direction.^30^ Trained with a systems framework, social workers connect individuals, organizations, and policy to address healthcare delivery challenges and gaps in services,^2,32^ which aligns with the competency of develop coalitions.^13^ Aligning with the system transformation competency, social workers can implement healthcare reform initiatives, and guide organizations and providers in the transition and adaptation to new structures,^33^ as illustrated with the implementation of virtual care and new innovations during the COVID-19 pandemic. The uneven distribution of social workers holding formal versus informal leadership roles in our study suggests that the leadership potential of social workers in primary care teams is being underutilized.

It is also important to highlight the value of social workers’ systemic and relational approaches to leadership in team-based contexts.^28,34,35^ The notion of “leaderful practice” moves away from a set of individual traits, behaviours, and powers and instead promotes leadership as a set of collective practices that many people carry out together to enact change.^36^ Leaderful practice is a social and collective process that can mobilize leadership potential in everyone.^36,37^ The notion of leaderful practice is particularly relevant to the team-oriented nature of work within primary care settings.^36,37^ This concept recognizes that anyone on the team can take on a leadership role, whether formally or informally. It is more about nurturing a set of skills around collective capacity, collaboration, concurrence, and compassion as a team.^36,37^ This is particularly important considering COVID-19 has underlined the importance of such leadership as a means of directing society toward change.^38^ Social work embraces a leadership practice approach by moving beyond what each individual profession can do and focuses on the capacity and vision of the collaborative team.^39^

This study highlights that social workers are carrying out leadership activities in primary care settings albeit informally. This informal and “unconscious” leadership is beneficial for the functioning of interprofessional primary care teams^19^ yet designated leadership roles enhances the capacity for those from interprofessional perspectives to facilitate team functioning.^21^ Reliance on informal leadership can be challenging for social workers, as it may be difficult to balance clinical and leadership demands simultaneously.^28^ Results from this study underscore the need for a supportive and sustainable organizational culture that recognizes, nurtures, and rewards interprofessional leadership. Organizational culture is linked to job satisfaction, and successful recruitment and retention of human resources^40,41^ which is of paramount importance during this crisis of worker shortage in primary care.^25,26^

In our study, social workers holding formal leadership positions self-reported high confidence in their leadership skills. It would be important to further explore what contributes to attaining high levels of confidence to target capacity development specifically for the primary care context. Education, training, and role modelling build capacity for social workers in other settings,^12,15,42^ yet need to be aligned with primary care contexts.^43^ Preparing social workers for leadership in primary care teams requires a greater emphasis on interprofessional approaches to education.^44^ Supervision and mentorship are cornerstones of professional development for social workers and are key factors for developing social workers’ confidence and capacity in leadership.^45,46,47^ Most social workers in this study had interest to further develop their leadership capacity. Primary care decision-makers are encouraged to implement a mechanism that enables access to systematic supervision and mechanisms for mentorship for social workers in primary care teams across Ontario and elsewhere. As primary care teams continue to expand, access to regular ongoing supervision for frontline social workers is necessary to facilitate ongoing leadership growth.^45,46,47^

One of the limitations of our study is the unequal distribution of participants in the formal versus informal subgroups, which may potentially skew interpretations. We conducted this study with social workers in primary care teams in Ontario, which may not be reflective of all jurisdictions. Our survey was conducted during a particular period, and it is anticipated that the experiences of social workers in primary care will evolve in relation to the pandemic.

## Conclusion

Leadership is needed to guide healthcare system changes, nurture patient-centred comprehensive care, and foster collaboration in interprofessional primary care teams. Social workers in primary care have leadership capacity, and are providing leadership to their primary care teams through formal and informal means. The leadership potential of social workers in primary care teams, however, is being underutilized and can be further developed.
